# Adsorption and Surface Analysis of Sodium Phosphate Corrosion Inhibitor on Carbon Steel in Simulated Concrete Pore Solution

**DOI:** 10.3390/ma15217429

**Published:** 2022-10-23

**Authors:** Ahmed Mohamed, Ulises Martin, David M. Bastidas

**Affiliations:** National Center for Education and Research on Corrosion and Materials Performance, NCERCAMP-UA, Department of Chemical, Biomolecular, and Corrosion Engineering, The University of Akron, 302 E Buchtel Ave, Akron, OH 44325-3906, USA

**Keywords:** corrosion inhibitor, phosphate, adsorption, density functional theory (DFT), electrochemistry, electrochemical impedance spectroscopy (EIS)

## Abstract

Corrosion of steel-reinforced concrete exposed to marine environments could lead to structural catastrophic failure in service. Hence, the construction industry is seeking novel corrosion preventive methods that are effective, cheap, and non-toxic. In this regard, the inhibitive properties of sodium phosphate (Na_3_PO_4_) corrosion inhibitor have been investigated for carbon steel reinforcements in 0.6 M Cl^−^ contaminated simulated concrete pore solution (SCPS). Different electrochemical testing has been utilized including potentiodynamic polarization, electrochemical impedance spectroscopy (EIS), and Mott-Schottky plots to test Na_3_PO_4_ at different concentrations: 0.05, 0.1, 0.3, and 0.6 M. It was found that Na_3_PO_4_ adsorbs on the surface through a combined physicochemical adsorption process, thus creating insoluble protective ferric phosphate film (FePO_4_) and achieving an inhibition efficiency (*IE*) up to 91.7%. The formation of FePO_4_ was elucidated by means of Fourier-transform infrared spectroscopy (FT–IR) and X-ray photoelectron spectroscopy (XPS). Quantum chemical parameters using density functional theory (DFT) were obtained to further understand the chemical interactions at the interface. It was found that PO_4_^3−^ ions have a low energy gap (Δ*E*_gap_), hence facilitating their adsorption. Additionally, Mulliken population analysis showed that the oxygen atoms present in PO4^3−^ are strong nucleophiles, thus acting as adsorption sites.

## 1. Introduction

Carbon steel rebars are often embedded in concrete structures to increase its tensile strength, which in turn enhances the structure durability and integrity. However, steel reinforcements may suffer degradation in the form of corrosion if exposed to a harsh marine environment, thus decreasing its service life. Cl^−^ ions ingress into the concrete matrix, and once above a critical concentration threshold, an autocatalytic acid hydrolysis reaction (see Equation (1)) will initiate causing a local pH drop, thus breaking down the passive film and initiating corrosion [1,2]. Consequently, oxyhydroxides will start forming on the surface of the rebar, increasing the crystallization pressure, and compromising the integrity of the concrete structure [3]. Different corrosion protection methods have been introduced to protect against corrosion in reinforced concrete; these include coatings, cathodic protection, the use of stainless steel rebars, and corrosion inhibitors. However, according to the literature, corrosion inhibitors are regarded as the most effective, economical, and easy to apply corrosion protective method [4,5].

(1) $${\mathrm{Fe}}^{2+}+{\;H}_{2}O+{\;\mathrm{Cl}}^{-}\to {\;\mathrm{FeOH}}^{+}+\mathrm{HCl}$$

One of the most popular corrosion inhibitors, particularly for reinforced concrete, are nitrites (NO_2_^−^), which are anodic corrosion inhibitors that oxidize ferrous ions forming a stable passive oxide film made up of maghemite (Fe_2_O_3_) and lepidocrocite (γ–FeOOH) [5,6]. However, recently NO_2_^−^ application has been limited in use due to ecological and environmental issues [5,7]. Some other alternatives include phosphate corrosion inhibitors as they are environmentally friendly [4]. The inhibitive property of phosphate compounds arises from their ability to react with the iron ions generated in the corrosion process, as well as with ionic species present in the mortar, such as Ca^2+^, which yields Ca_3_(PO_4_)_2_ precipitates, filling the pores and cracks of the mortar, thus hindering the diffusion of aggressive ions (i.e., Cl^−^) [8]. Bastidas et al. tested three phosphate-based corrosion inhibitors (Na_3_PO_4_, Na_2_HPO_4_, and Na_2_PO_3_F) on carbon-steel-reinforced ordinary Portland cement mortar matrices. It was concluded that the three phosphates acted as effective anodic corrosion inhibitors forming a protective FePO_4_ compact layer [4].

Similarly, the inhibition mechanism of phosphates in simulated concrete pore solution (SCPS) is due to the incorporation of phosphate ions in the corrosion oxide layer, thus forming a stable passive film made up of iron phosphate [8,9]. A study investigated the inhibition performance of Na_3_PO_4_ in SCPS at different inhibitor-to-chloride ratios, 0.2 and 0.6 [9]. It was concluded that after 90 days, high concentrations of phosphates were able to mitigate pitting, while low concentrations just delayed the initiation of the localized attack [9]. Moreover, it was concluded that Na_3_PO_4_ acted as a mixed corrosion inhibitor, forming a stable double-layered structured passive oxide/phosphate film [9]. In another study, phosphate-based corrosion inhibitors were classified as cathodic, as it was found that it precipitates on cathodic sites of the working electrode [10]. One hypothesis could be that at a low inhibitor-to-chloride ratio, phosphates act as a cathodic corrosion inhibitor, while at high ratios it acts as a mixed one [10]. As it can be seen, there are contradicting opinions in the literature on the classification of phosphate corrosion inhibitors and their inhibition mechanism.

The use of quantum computational chemistry by applying density functional theory (DFT) can help to understand and articulate the inhibition mechanism of different corrosion inhibitors [11]. DFT analysis provides correlation between the effect of molecular structure on the inhibition and the adsorption process, which include the effect of electronic configuration, π-bonds, heteroatoms, and heterocycles [12]. Usually, the frontier molecular orbitals, dipole moments, Mulliken population analysis, and other quantum chemical properties contribute towards identifying the active reaction sites of a corrosion inhibitor [11]. Consequently, DFT calculations will be utilized to elucidate the physicochemical mechanism and reactivity of Na_3_PO_4_.

In this work, the corrosion inhibition mechanism of Na_3_PO_4_ in 0.6 M Cl^−^ SCPS will be investigated by means of electrochemical testing using potentiodynamic polarization (PDP), electrochemical impedance spectroscopy (EIS), and Mott-Schottky plots to study the semiconductive properties of the passive film. Four different concentrations of Na_3_PO_4_ will be tested to understand its effect and find the adsorption isotherm occurring at the interface. Additionally, density functional theory (DFT) modeling will be used to elucidate quantum chemical properties and correlate them to the inhibition performance of Na_3_PO_4_ corrosion inhibitor. Optical microscopy (OM), infinite focus microscopy (IFM), Fourier-transform infrared spectroscopy (FT–IR), and X-ray photoelectron spectroscopy (XPS) will be used to comprehensively analyze the surface of carbon steel samples in the presence of Na_3_PO_4_ to unravel and confirm its inhibition mechanism.

## 2. Materials and Methods

Carbon steel samples were used as the working electrode for electrochemical testing in this study, having an exposed surface area of 6 cm^2^. An electrical connection was established by attaching a copper wire to the surface. The samples were degreased, washed, and cleaned with deionized water, ethanol, and acetone to remove any surface contaminants. The elemental composition of the carbon steel samples can be seen in Table 1.

Electrochemical tests were carried out in an SCPS prepared by filtering a saturated calcium hydroxide (Ca(OH)_2_) solution. Accordingly, 0.6 M Cl^−^ were added by means of NaCl to mimic concrete structures exposed to harsh marine environments [12]. The pH of the solution was measured at room temperature and found to be 12.6. Different concentrations of Na_3_PO_4_ corrosion inhibitor were added to test its inhibition efficiency (*IE*): 0.05, 0.1, 0.3, and 0.6 M Na_3_PO_4_. All solutions were prepared from analytical grade reagents and deionized water.

**Figure 1:**
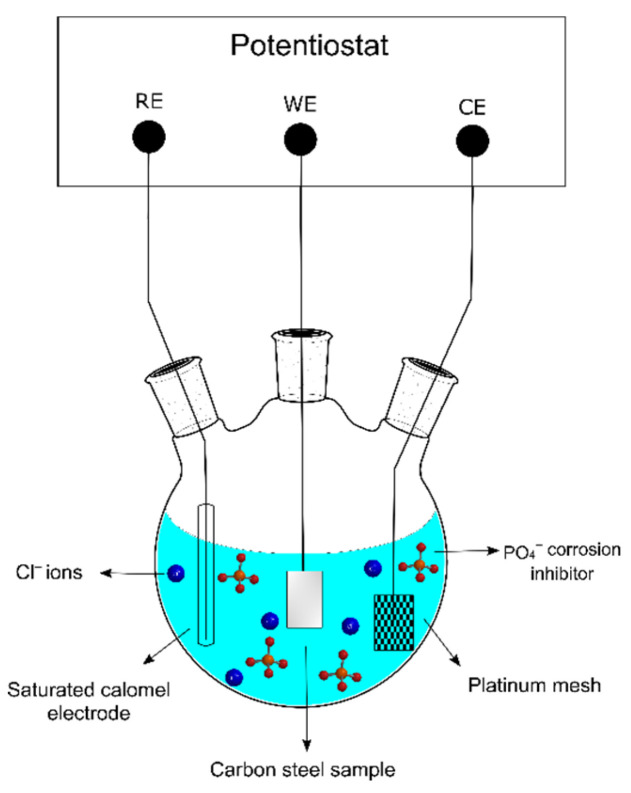
Schematic of the three-electrode configuration electrochemical cell setup of carbon steel in 0.6 M Cl^−^ SCPS with the dissociated PO_4_^3−^ corrosion inhibitor.

Several electrochemical measurements including PDP, EIS, and Mott–Schottky were conducted using a Gamry series 600 potentiostat with a temperature-controlled three-electrode configuration setup. The working electrode (WE) was a carbon steel sample, the counter electrode (CE) was a platinum mesh, and the reference electrode (RE) was a Ag/AgCl (SSC) electrode. The electrochemical test setup can be seen in Figure 1.

Initially, an open circuit potential (OCP) was monitored until a steady-state value was achieved. After this, an EIS analysis was conducted at the OCP in the frequency range between 10^5^ and 10^−2^ Hz with a 10 mV AC excitation signal at the rate of 5 steps/decade, following the ASTM G106-89 standard [13]. Consequently, a PDP was performed in the scan range from −200 mV_OCP_ to 200 mV_OCP_ in accordance with ASTM G61-86 [14]. Furthermore, to evaluate the semiconductive properties of the passive film formed on Na_3_PO_4_ inhibited carbon steel samples, a Mott–Schottky analysis was performed from −400 mV_SCC_ to +500 mV_SCC_ using a potential step of 25 mV. Finally, the adsorption isotherm of Na_3_PO_4_ corrosion inhibitor was identified by correlating the *IE* to the corrosion inhibitor concentration. It should be noted that all electrochemical tests were performed in triplicates to ensure reproducibility.

Different surface characterization techniques were utilized to elucidate and understand the adsorption of Na_3_PO_4_ on carbon steel electrodes, these include OM, IFM, FTIR, and XPS. The surface of the carbon steel sample after electrochemical testing was investigated by OM using a Nikon eclipse MA 100 metallographic microscope. Furthermore, an IFM analysis was performed by means of an Alicona Infinite Focus G5 Microscope to analyze the surface topology of the uninhibited (i.e., blank) and Na_3_PO_4_ inhibited carbon steel samples. The FTIR surface characterization was performed on the tested carbon steel samples using a PerkinElmer Fourier transform infrared spectrometer. Finally, the XPS analysis was performed using a PHI 5000 VersaProbe II X-ray photoelectron spectrometer with a take-off angle of 45°, voltage excitation signal of 15 kV, residual pressure less than 10^−6^ Pa, and a power of 25 W. Surface contaminants were removed, up to 2 nm, using an Ar ion bombardment and the calibration process was performed on a Ag substrate, using separate measurements for the Ag 3d_5/2_ peak found at 368.3 eV. The high-resolution XPS peaks were fitted using a Gaussian-Lorentzian function.

Quantum chemical properties were calculated using DFT, which was performed using Spartan version 8.0 software. The molecule geometry was fully optimized employing a B3LYP functional, and using a 6–31G (d,p) basis set to determine the energy of the highest occupied energy molecular orbital (*E*_HOMO_), energy of the lowest occupied energy molecular orbital (*E*_LUMO_), energy gap (Δ*E*_gap_), dipole moment (*µ*_D_), electrostatic potential mapping, and Mulliken charges.

## 3. Results and Discussion

**Figure 2:**
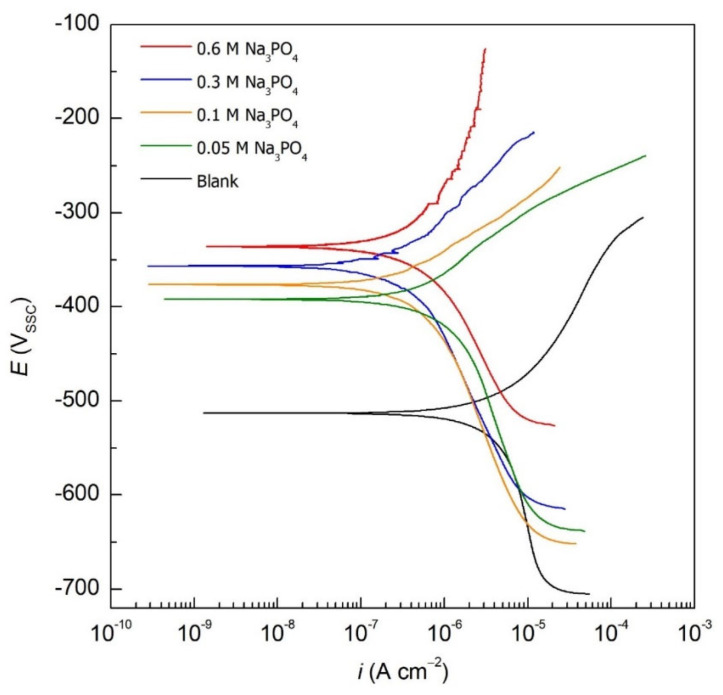
PDP curves for carbon steel samples in the absence and presence of different concentrations of Na_3_PO_4_ in 0.6 M Cl^−^ SCPS at 25 °C.

Figure 2 illustrates the PDP curves of the blank and different concentrations of Na_3_PO_4_ inhibited carbon steel samples in 0.6 M Cl^−^ contaminated SCPS at 25 °C. The electrochemical corrosion kinetics were obtained by extrapolating the linear Tafel segment of the anodic and cathodic branches of the PDP curves. Such parameters are presented in Table 2 and include corrosion potential (*E*_corr_), corrosion current density (*i*_corr_), and anodic (*β*_a_) and cathodic (*β*_c_) Tafel slopes. The *IE* and surface coverage (*θ*) are also presented in Table 2 and calculated using Equations (2) and (3):

(2) $$IE\;\left(\%\right)=\left(1-\frac{i_{\mathrm{corr}}}{i_{\mathrm{corr},b}}\right)\times 100$$

(3) $$\theta =\frac{i_{\mathrm{corr},b}-i_{\mathrm{corr}}}{i_{\mathrm{corr},b}}$$

where *i*_corr_ and *i*_corr,b_ are the corrosion current densities of the inhibited and blank carbon steel samples, respectively. As seen in Table 2, Na_3_PO_4_ imparts an anodic corrosion inhibition mechanism, thus showing an ennoblement of the *E*_corr_ values from −514 mV_SSC_ up to −390, −341, −378, and −371 mV_SSC_ for 0.05, 0.1, 0.3, and 0.6 M Na_3_PO_4_, respectively. This indicates that the iron dissolution half-reaction (anodic reaction) is being inhibited by Na_3_PO_4_ by forming a protective barrier layer that hinders corrosion. This anodic inhibition mechanism can be further evidenced by the significant change in the *β*_a_ of the Na_3_PO_4_ inhibited samples relative to the blank. The presence of Na_3_PO_4_ was able to decrease the *i*_corr_ at every concentration achieving an *IE* of 80.8, 87.5, 90.4, bs 91.7% for 0.05, 0.1, 0.3, and 0.6 M Na_3_PO_4_ inhibited samples, respectively. This great anticorrosion performance is attributed to the ability of phosphates to compete with Cl^−^, reacting with Fe ions forming stable insoluble compounds, hence protecting the surface of the working electrode [9]. The presence of Na_3_PO_4_ allows the formation of a 3D double-layered surface passive film layer made up of iron oxides and insoluble iron phosphate (FePO_4_)—a characteristic of interphase corrosion inhibition [15,16]. Increased concentrations of Na_3_PO_4_ allow a compact passive film to be developed, hence achieving better corrosion inhibition performance, as seen in Table 2.

**Figure 3:**
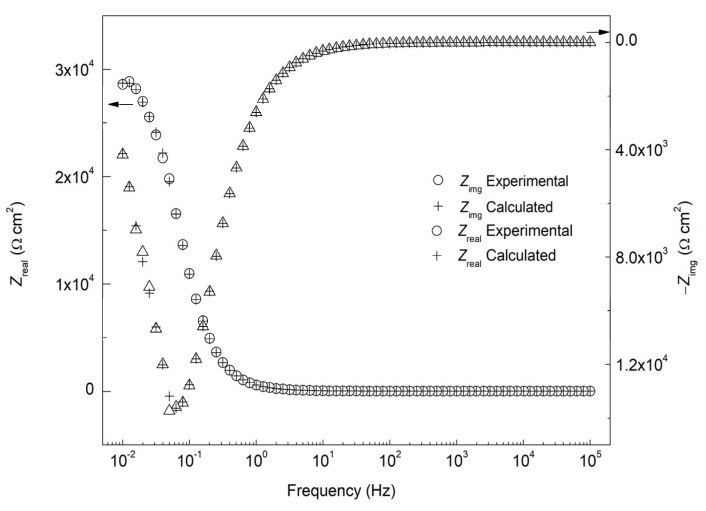
Kramers-Kronig (KK) transforms of 0.6 M Na_3_PO_4_ inhibited carbon steel sample in 0.6 M Cl^−^ SCPS at 25 °C.

EIS electrochemical testing was performed to understand the film formation and interphase inhibition of Na_3_PO_4_ at the electrode/electrolyte interface. After recording the EIS plots, the validity and robustness of the EIS data was evaluated by using the Kramers−Kronig (KK) transforms, defined in Equations (4) and (5) [7]:

(4) $$Z_{\mathrm{real}}\left(\omega \right)=Z_{\mathrm{real}}\left(\infty \right)-\left(\frac{2}{\pi }\right)\int_{o}^{\infty }\frac{xZ_{\mathrm{img}}\left(x\right)-\omega Z_{\mathrm{img}}\left(\omega \right)}{x^{2}-\omega^{2}}dx$$

(5) $$Z_{\mathrm{img}}\left(\omega \right)=-\left(\frac{2\omega }{\pi }\right)\int_{o}^{\infty }\frac{Z_{\mathrm{real}}\left(x\right)-Z_{\mathrm{real}}\left(\omega \right)}{x^{2}-\omega^{2}}dx$$

where *Z*_real_, *Z*_img_, ω, and *x* are the real impedance, imaginary impedance, frequency of the transform, and frequency of integration, respectively [17,18]. From the experimental and calculated impedance data values, the validity and robustness of the EIS can be verified. Figure 3 shows that the KK transforms for the 0.6 M Na_3_PO_4_ inhibited carbon steel sample in 0.6 M Cl^−^ SCPS at 25 °C, where the experimental data are denoted as symbols, and the calculated ones with crosses. The consistency between calculated and experimental EIS data shows the robustness of the obtained experimental results.

**Figure 4:**
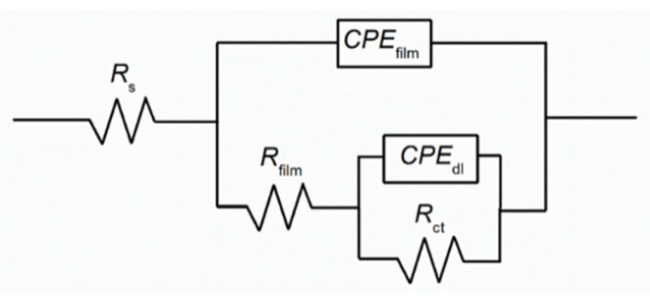
Electrical equivalent circuit (EEC) used to fit EIS data.

The EIS results were fitted to an electrical equivalent circuit (EEC), as seen in Figure 4, showing a hierarchy-distributed circuit where *R*_s_, *R*_film_, and *R*_ct_ are the solution, passive film, and charge transfer resistances, respectively. Two-time constants were added (*R−CPE*) indicating two relaxation processes where one constant phase element (*CPE*) corresponds to the passive film formed (intermediate frequencies) and the other to the electrochemical double layer interface (low frequencies).

**Figure 5:**
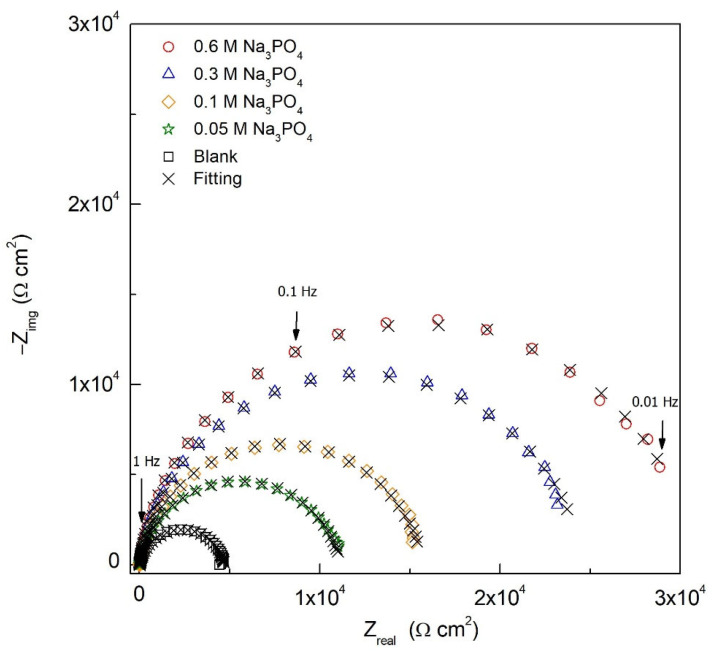
Nyquist plots for carbon steel samples in the absence and presence of different concentrations of Na_3_PO_4_ in 0.6 M Cl^−^ SCPS at 25 °C.

As seen in Figure 5, a sound agreement was established between the proposed EEC and EIS experimental data having a low chi-squared value of 10^−4^ and a percentage error of each electrochemical parameter below 10%. Table 3 shows all the fitted data, where *Y*_film_, *Y*_dl_, *n*_film_, and *n*_dl_ are the admittance for the passive film, the admittance for the double layer, the *CPE* exponent of the film layer, and the *CPE* exponent of the double layer, respectively. The values of *n*_film_ or *n*_dl_ can range from 0 to 1, where 1 indicate an ideal capacitor, 0 an ideal resistor, and *n* < 1 a behavior associated with surface heterogeneity and defects [19].

The *R*_s_ for the inhibited and blank samples are relatively similar, ranging from 5.98 to 8.89 Ω cm^2^. The *R*_film_, on the other hand, increases with the increasing concentrations of Na_3_PO_4_ reaching an order of magnitude greater for the 0.1, 0.3, and 0.6 M Na_3_PO_4_ inhibited carbon steel samples. This indicates the formation of a protective compact passive film formed by ferrous and ferric phosphate compounds, Fe_3_(PO_4_)_2_ and FePO_4,_ respectively [9,20]. This protective passive film effect can also be seen in the change of the *R*_ct_, where Na_3_PO_4_ inhibited samples exhibited an order of magnitude increase, relative to the blank, at all concentrations achieving an IE of 68.6, 77.6, 85.4, and 88.6% for 0.05, 0.1, 0.3, and 0.6 M Na_3_PO_4_, respectively, corroborating the PDP results. An increased *R*_ct_ indicates a hindered charge transfer process between the electrode/electrolyte interface due to the formation of the protective passive film [7,20].

The effective capacitance of the electrochemical double layer (*C*_eff,dl_) and passive film (*C*_eff,film_) were calculated for the blank and Na_3_PO_4_ inhibited samples using the equations provided by Brug et al. and Mansfeld et al., respectively (Equations (6) and (7)) [21,22]. Additionally, the effective passive film thickness (*d*_eff,film_) was calculated using Equation (8) [21,23].

(6) $$C_{\mathrm{eff},\mathrm{dl}}=Y_{\mathrm{dl}}{}^{\frac{1}{n_{\mathrm{dl}}}}{\left(\frac{1}{R_{s}}-\frac{1}{R_{\mathrm{ct}}}\right)}^{\left(\frac{n_{\mathrm{dl}}-1}{n_{\mathrm{dl}}}\right)}$$

(7) $$C_{\mathrm{eff},\mathrm{film}}=Y_{\mathrm{film}}{\left(\omega_{m}^{\prime \prime }\right)}^{n_{\mathrm{film}\;}-1}$$

(8) $$d_{\mathrm{eff},\mathrm{film}}=\frac{\varepsilon_{o}\;\varepsilon_{\mathrm{film}}}{C_{\mathrm{eff},\mathrm{film}}}$$

where *ω*_m_″ corresponds to the frequency where the maximum imaginary impedance value is achieved, *ε*_film_ is the dielectric constant of the oxide film (a value of 30 was used [24]), and *ε*_o_ is the vacuum permittivity (8.85 ×10^−14^ F cm^−1^).

**Figure 6:**
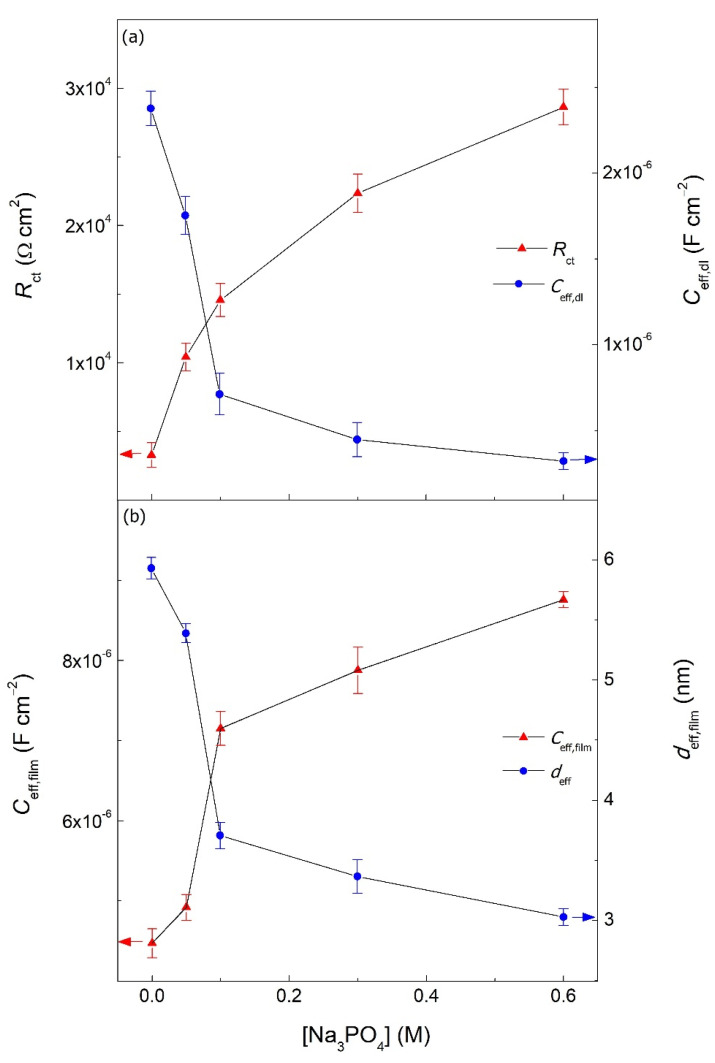
Electrochemical parameters obtained using EIS analysis for the blank and Na_3_PO_4_ inhibited carbon steel sample in 0.6 M Cl^−^ contaminated SCPS at 25 °C: (**a**) electrochemical double layer charge transfer resistance (*R*_ct_) and effective capacitance(*C*_eff,dl_), and (**b**) passive oxide film effective capacitance (*C*_eff,film_) and film thickness (*d*_eff,film_).

Table 4 shows all the quantitative values of *C*_eff,dl_, *C*_eff,film_, and *d*_eff,film_ for the blank and Na_3_PO_4_ inhibited carbon steel samples in 0.6 M Cl^−^ contaminated SCPS at 25 °C. *C*_eff,dl_ decreased in the presence of Na_3_PO_4_ corrosion inhibitor relative to the blank at every concentration reaching an order of magnitude lower for the 0.1, 0.3, and 0.6 M Na_3_PO_4_ inhibited samples. The decrease in the *C*_eff,dl_ is due to the dielectric constant of water molecule being larger than that of the corrosion inhibitor. In the presence of Na_3_PO_4_, the water molecules at the metal/electrolyte interface will be replaced by the dissociated phosphate ions having a lower dielectric constant and thus a lower *C*_eff,dl_ [25]_._ Figure 6a shows the change in *R*_ct_ and *C*_eff,dl_ for the blank and Na_3_PO_4_ inhibited samples, where the variations between them are in sound agreement showing that the highest achieved *R*_ct_ corresponds to the lowest *C*_eff,dl_, thus suggesting the formation of a stable and protective passive film [26,27].

Figure 6b illustrates the variation between the *C*_eff,film_ and *d*_eff,film_ for the blank and Na_3_PO_4_ in 0.6 M Cl^−^ contaminated SCPS at 25 °C. The *C*_eff,film_ increased with increasing concentrations of Na_3_PO_4_, hence producing a thinner passive film; a characteristic attributed to the nature of interphase corrosion inhibitors producing a more compact 3D interphase passive film (i.e., ferric phosphate) [15,28,29]. This is in accordance to previously reported results found in the literature, hence corroborating electrochemical results [9,30]. However, to further understand this interphase inhibitive mechanism of Na_3_PO_4_, the semiconducting properties of the film needs be to be analyzed and studied using Mott−Schottky plots.

**Figure 7:**
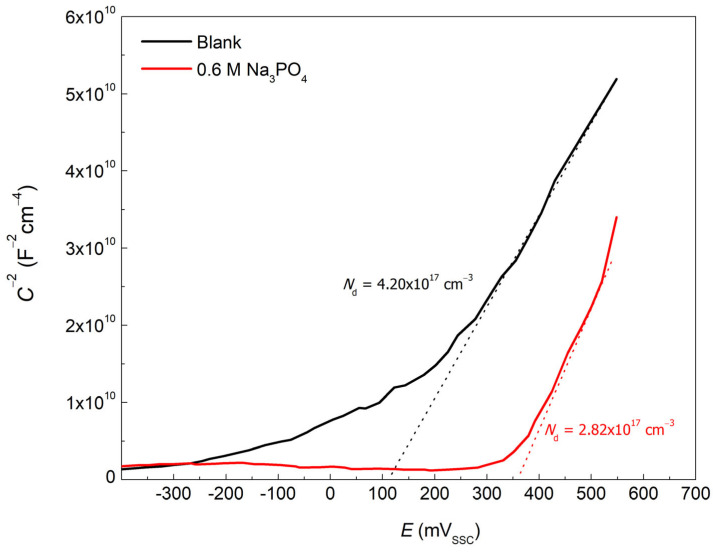
Mott−Schottky plots for the blank and 0.6 M Na_3_PO_4_ inhibited carbon steel sample in 0.6 M Cl^−^ contaminated SCPS at 25 °C.

Figure 7 shows the Mott−Schottky plots of the blank and 0.6 M Na_3_PO_4_ carbon steel inhibited samples in 0.6 M Cl^−^ contaminated SCPS at 25 °C, relating the space charge capacitance (*C*) to the applied potential (*E*). *N*_d_, the number of donor defects, is related to the number of point defects (either metal interstitials and/or oxygen vacancies) present in the passive film and can be determined using Equations 9 and 10 [20,31,32]:

(9) $$\frac{1}{C^{2}}=\frac{2}{\varepsilon_{0}\varepsilon qN_{d}}\left(E-E_{FB}-\frac{KT}{q}\right)$$

(10) $$N_{d}=\frac{2}{\varepsilon \varepsilon_{o}qm}$$

where *q* denotes the charge of electron (1.602 ×10^−19^ C), *T* is the absolute temperature, *k* is the Boltzmann constant (1.38 ×10^−23^ J K^−1^), *E*_FB_ is the flat band potential, and *m* is the slope of the Mott–Schottky curve.

The positive slopes observed in Figure 7 are indications of the *n*-type semiconductor behavior of carbon steel, suggesting an electron donor carrier in the space charge between the oxide film and the electrolyte interface [33,34]. The *N*_d_ of the blank and 0.6 M Na_3_PO_4_ inhibited samples, calculated using Equation (10), was found to be 4.20 ×10^17^ cm^−3^ and 2.82 ×10^17^ cm^−3^, respectively. The blank showed a higher *N*_d_ indicating an increased number of defects in the passive film, thus allowing the adsorption of Cl^−^ ions on the surface and initiating corrosion [35]. In contrast, Na_3_PO_4_ was able to lower the number of defects (*N*_d_ = 2.82 ×10^17^ cm^−3^) creating a more orderly and compact passive film protecting the sample from the corrosive environment. This behavior is consistent with the *R*_film_ found in the EIS fitting, since increased concentrations of Na_3_PO_4_ produced higher *R*_film_ compared to the blank. Therefore, it shows the inhibitive properties of Na_3_PO_4_ in producing an orderly, compact, and protective passive film made up of insoluble phosphate compounds. To better understand this adsorption process and inhibition mechanism, the adsorption isotherms of Na_3_PO_4_ have been studied.

Adsorption isotherms provide insights into the physicochemical interactions between the adsorbed corrosion inhibitor and the metal substrate at the interface level by studying the adsorption equilibrium constant (*K*_ads_) and Gibbs free energy of adsorption (Δ*G*^°^_ads_) through correlating the surface coverage (*θ*) to the concentration of the adsorbed species [36,37]. The adsorption of Na_3_PO_4_ will be studied utilizing the PDP results found in Table 2. Different adsorption isotherms have been tested including Langmuir, Temkin, Freundlich, Frumkin, and El-Awady; the best fit was obtained through the Langmuir adsorption isotherm, as seen in Table 5 [37].

**Figure 8:**
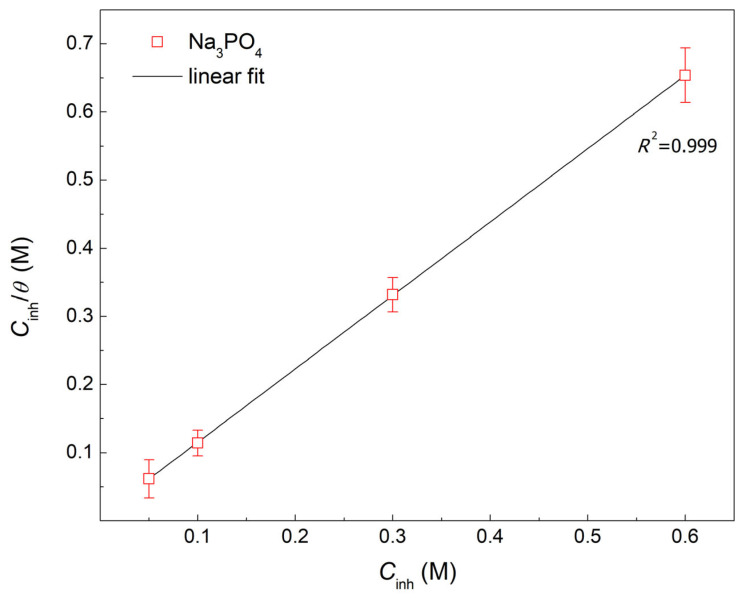
Langmuir adsorption isotherm for Na_3_PO_4_ in 0.6 M Cl^−^ contaminated SCPS at 25 °C.

Figure 8 shows the Langmuir adsorption isotherm of Na_3_PO_4_ in 0.6 M Cl^−^ SCPS at 25 °C having a correlation coefficient of 0.999, indicating that the adsorption of Na_3_PO_4_ follows the Langmuir model. Accordingly, the *K*_ads_ was obtained from the *y*-intercept and calculated to be 131 M^−1^, while Δ*G*^°^_ads_ was calculated using Equation (11) [38]:

(11) $$\Delta G_{\mathrm{ads}}^{{}^{\circ}}=-RT\;\ln\;\left(55.5K_{\mathrm{ads}}\right)$$

where *R* is the universal gas constant and 55.5 is the molar concentration of water. The calculated Δ*G*^°^_ads_ was found to be −22 kJ/mol and can used as a criterion to classify the mode of adsorption. According to the literature, a Δ*G*^°^_ads_ lower than −20 kJ/mol signifies a physisorption process where the inhibitor adsorbs on the surface of the substrate though electrostatic interactions [39,40,41]. In contrast, a Δ*G*^°^_ads_ less than −40 kJ/mol signifies a chemisorption process where the inhibitor adsorbs through electron transfer and creating a feedback bond with the metal atom [39,40,41]. A −40 kJ/mol < Δ*G*^°^_ads_ < −20 kJ/mol indicates a mixed adsorption mechanism of simultaneous chemical and physical adsorption [39,40,41]. Accordingly, the adsorption of Na_3_PO_4_ is a spontaneous mixed mechanism where both electrostatic interaction and electron transfer occur during the inhibition process. The electrostatic interaction origins from the near surface oxygen vacancies attracting the dissociated phosphate ions. Consequently, the chemical interaction happens during the electron transfer to the unoccupied *d*−orbitals of the metal cation at the surface of the working electrode, creating the insoluble phosphate compound film.

The inhibition mechanism of Na_3_PO_4_ arises from its ability to dissociate into PO_4_^3−^ ions, competing with Cl^−^ and adsorbing physically and chemically forming insoluble iron phosphate compounds on the surface, Fe_3_(PO_4_)_2_ and FePO_4_ [8,9,10]. The presence of Na_3_PO_4_ initiates the precipitation of ferrous phosphate through a dissolution precipitation mechanism shown in Equation (12) [9]:

(12) $$3\mathrm{Fe}+2{\mathrm{PO}}_{4}^{3-}\to {\mathrm{Fe}}_{3}{\left({\mathrm{PO}}_{4}\right)}_{2}+6e^{-}$$

The PO_4_^3−^ ions will adsorb on the surface of the working electrode creating a double-layered structure, where the inner layer will be made up of Fe_3_O_4_ and/or Fe_2_O_3_, while the outer one will be Fe_3_(PO_4_)_2_ which will gradually oxidize to form FePO_4_ [8,9]. The formation of this protective ferric phosphate is thermodynamically favored over the formation of iron chloride (FeCl_2_ and FeCl_3_), thus creating a protective, stable, and compact passive film [4,8]. The different chemical reactions of iron phosphate and iron chloride, along with their Gibbs free energy of formation (Δ*G*^°^_f_) are presented in the following Equations (13)–(16) [4,8]:

(13) $$3{\mathrm{Fe}}^{2+}+2{\mathrm{PO}}_{4}{}^{3-}⇆{\mathrm{Fe}}_{3}{\left({\mathrm{PO}}_{4}\right)}_{2};\;\;\Delta G_{f}^{{}^{\circ}}=-2444.80\;\mathrm{kJ}/\mathrm{mol}$$

(14) $${\mathrm{Fe}}^{3+}+{\mathrm{PO}}_{4}{}^{3-}⇆{\mathrm{FePO}}_{4};\;\;\Delta G_{f}^{{}^{\circ}}=-1663.98\;\mathrm{kJ}/\mathrm{mol}$$

(15) $${\mathrm{Fe}}^{2+}+2{\mathrm{Cl}}^{-}⇆{\mathrm{FeCl}}_{2};\;\;\Delta G_{f}^{{}^{\circ}}=-302.35\;\mathrm{kJ}/\mathrm{mol}$$

(16) $${\mathrm{Fe}}^{3+}+3{\mathrm{Cl}}^{-}⇆{\mathrm{FeCl}}_{3};\;\;\Delta G_{f}^{{}^{\circ}}=-668.11\;\mathrm{kJ}/\mathrm{mol}$$

**Figure 9:**
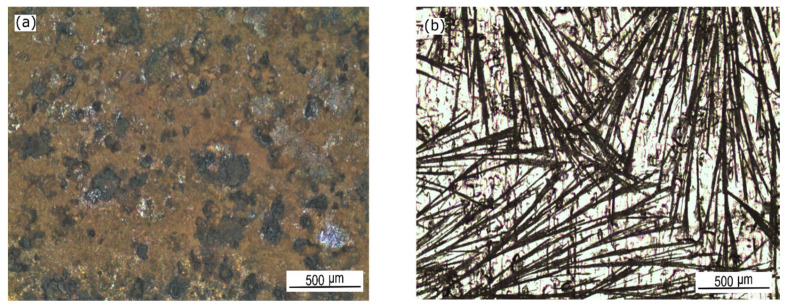
Optical microscope (OM) micrograph for the (**a**) blank, and (**b**) 0.6 M Na_3_PO_4_ inhibited carbon steel sample in 0.6 M Cl^−^ SCPS at 25 °C.

This inhibitive property of Na_3_PO_4_ can be observed by OM micrographs of carbon steel samples in the presence and absence of 0.6 M Na_3_PO_4_ in 0.6 M Cl^−^ SCPS at 25 °C (see Figure 9). The blank showed extensive corrosion products due to Cl^−^ attacks, thus breaking down the passive film and creating iron oxy/hydroxides. In contrast, the 0.6 M Na_3_PO_4_ inhibited carbon steel sample shows clear indications of ferric phosphate created by the dissolution precipitation mechanism, mentioned previously [9]. The formed passive film was able to protect the working electrode from the Cl^−^ attacks, thus hindering the iron acid hydrolysis reaction (see Equation (1)) and preventing the initiation of the corrosion process. It should be noted that PO_4_^3−^ can also react with Ca(OH)_2_ in the SCPS forming the low soluble Ca_3_(PO_4_)_2_ precipitate [42].

**Figure 10:**
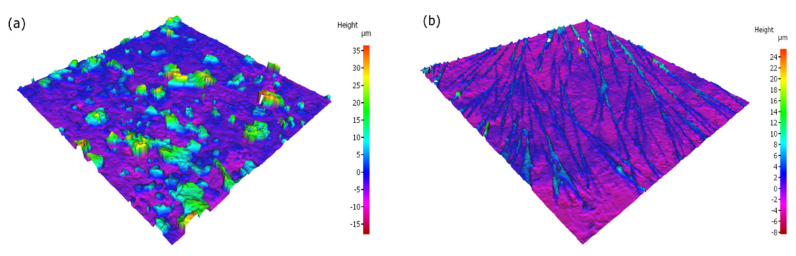
IFM surface analysis of carbon steel samples after electrochemical testing of the (**a**) blank, and (**b**) 0.6 M Na_3_PO_4_ corrosion inhibitor in 0.6 M Cl^−^ SCPS at 25 °C.

IFM surface analysis can be seen in Figure 10 for the carbon steel samples in 0.6 M Cl^−^ SCPS in the absence and presence of 0.6 M Na_3_PO_4_ at 25 °C after electrochemical testing. The blank exhibits a rough surface reaching heights of 30 µm, signifying the buildup of corrosion products; also, a max of 10 µm depth was observed which is attributed to the extensive dissolution process of the carbon steel sample in this aggressive environment. In contrast, the 0.6 M Na_3_PO_4_ exhibits a smoother surface due to the formation of the compact ferric phosphate film; some peaks are observed reaching up to 7 µm due to the dissolution precipitation mechanism of Na_3_PO_4_. However, further surface analysis will be needed to understand the composition of the passive film created.

**Figure 11:**
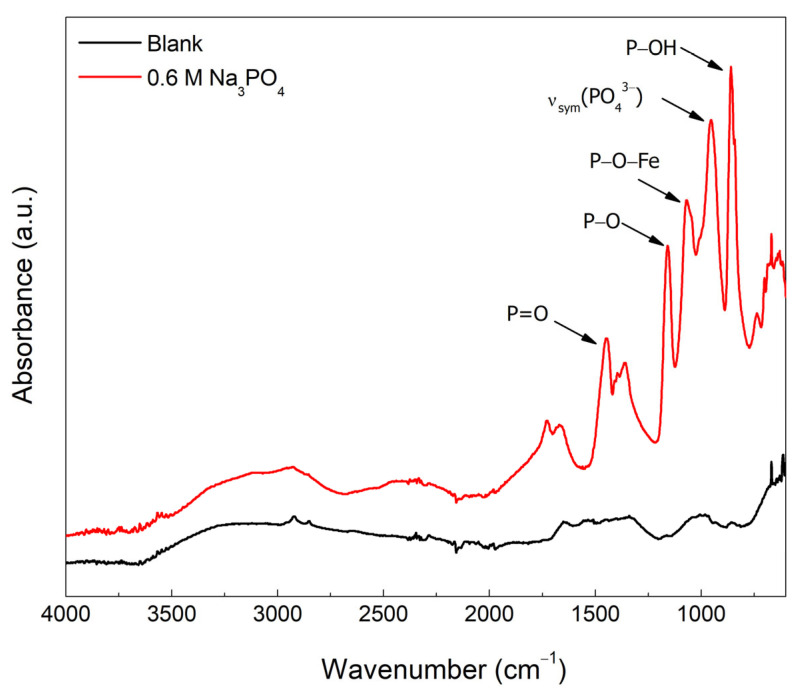
FT−IR spectrum for the blank and 0.6 M Na_3_PO_4_ inhibited carbon steel sample in 0.6 M Cl^−^ contaminated SCPS at 25 °C.

FTIR was used to analyze the surface composition of the blank and 0.6 M Na_3_PO_4_ inhibited carbon steel samples in 0.6 M Cl^−^ SCPS at 25 °C, shown in Figure 11. The Na_3_PO_4_ inhibited sample show distinctive peaks that is absent in the blank, which elucidates the adsorption of phosphate on the surface. The peak around 863 cm^−1^ is attributed HPO_4_^2−^ since this species will coexist with PO_4_^3−^ at pH~12.6, which is in the range of the pKa_2_ = 12.3 [8,43]. Peaks at 955, 1066, 1154, and 1359 cm^−1^ are representative to ν_sym_ (PO_4_^3−^), P−O−Fe, P−O, and P=O bond stretching vibrations, respectively, confirming the adsorption of phosphate ions creating insoluble ferric phosphate compounds seen in the P−O−Fe peak [43,44,45,46].

**Figure 12:**
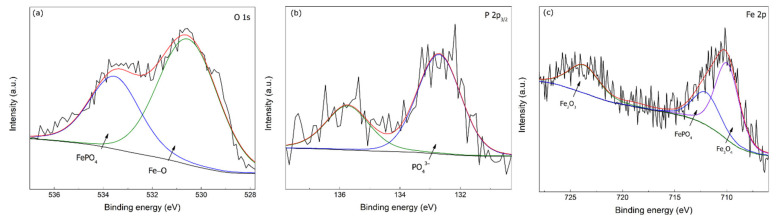
High-resolution XPS spectra for 0.6 M Na_3_PO_4_ inhibited carbon steel sample in 0.6 M Cl^−^ contaminated SCPS at 25 °C: (**a**) O 1s, (**b**) P 2p_2/3_, and (**c**) Fe 2p.

Moreover, the high-resolution XPS spectra for O 1s, P 2p_3/2_, and Fe 2p are illustrated in Figure 12 for 0.6 M Na_3_PO_4_ in 0.6 M Cl^−^ SCPS at 25 °C. Two peaks were used to split the high-resolution spectra for O 1s, one at 533.60 and 530.56 eV corresponding to FePO_4_ and Fe−O, respectively, evidencing the formation of ferric phosphate passive film [8,47,48]. Furthermore, the P 2p_2/3_ had two distinctive peaks: one representative of PO_4_^3−^ (132.70 eV) corresponding to FePO_4_ and the other at 135.75 eV, which is a peak usually associated with pure Na_3_PO_4_ [49]. Finally, the Fe 2p was split into three peaks at 723.90, 712.2, and 710.07 eV corresponding to Fe_2_O_3_, FePO_4_, and Fe_3_O_4_, thus confirming the formation of a double-layered passive film as mentioned previously [8,35,50]. It should be noted that some variations in the peak values can occur due to the chemical composition of the electrolyte.

**Figure 13:**
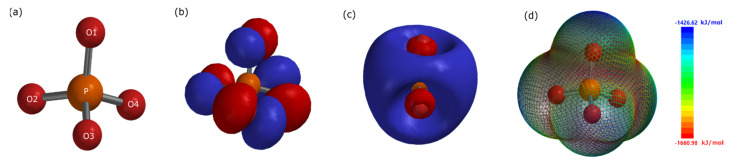
DFT quantum chemical calculations for the dissociated PO_4_^3−^ ion. (**a**) Optimized structure of the PO_4_^3−^ ion, (**b**) HOMO, (**c**) LUMO, and (**d**) electrostatic potential mapping. Red and blue orbitals represent positive and negative orbital spins, respectively.

Computational chemistry, nowadays, has become an important factor in understanding the inhibition mechanism at the metal interface [11]. Quantum chemical properties can assess the reactivity and adsorption sites of corrosion inhibitors, correlating the molecular structure to the inhibition process. In this regard, different quantum chemical properties were calculated for the optimized structure of the dissociated PO_4_^3−^ ion. The geometry optimization of the phosphate ion was carried out using DFT with Becke’s three parameter hybrid functional and the Lee−Yang− Parr correlation (B3LYP)/6–31G (d,p) to find the *E*_HOMO_, *E*_LUMO_, Δ*E*_gap_, *µ*_D_, Mulliken charges, and electrostatic potential mapping of the dissociated PO_4_^3−^ ion, shown in Figure 13 and Table 6 and Table 7.

*E*_HOMO_ (−2.88 eV) provides insight of the ability of a molecule to donate an electron to vacant cation orbital, while *E*_LUMO_ (5.76 eV) indicates the ability of a structure to accept an electron creating a feedback bond [12,51]. A high *E*_HOMO_ along with a low *E*_LUMO_ are often associated with enhanced ability of the corrosion inhibitor to adsorb onto the metal surface, thus imparting superior corrosion inhibition. The difference between *E*_HOMO_ and *E*_LUMO_ would be the Δ*E*_gap_ (*E*_LUMO_ − *E*_HOMO_ = 8.64 eV), which gives an indication of the reactivity of a molecule [12,52]. A low Δ*E*_gap_ is favorable since low energy will be required to put the molecule in an excited stage, thus affecting the chemical reactivity, kinetic stability, and polarizability of a molecule [53]. As seen in Table 6, the dissociated PO_4_^3−^ has a low Δ*E*_gap_ (i.e., reactive) indicating that it will be able to compete with Cl^−^ ions and adsorb on the metal interface, thus creating a protective film and achieving an *IE* of 91.7%. Previous studies demonstrated a positive correlation between low Δ*E*_gap_ and inhibition performance, indicating that corrosion inhibitors possessing lower Δ*E*_gap_ often perform superior to ones with high Δ*E*_gap_ [11]. However, it should be noted that there are many different factors that affect the inhibition process, and these are not absolute rules, just mere indications. The Mulliken charges along with the electrostatic mapping were calculated to identify the reaction/adsorption sites of PO_4_^3−^ corrosion inhibitor with the metal surface. Finally, the *µ*_D_ have been calculated and found to be 1.79 Debye, which measures the overall polarity of the inhibitor; nevertheless, there are contradicting opinions in the literature on the actual correlation between the dipole moment and inhibition performance [54,55].

As seen in Figure 13b, the *E*_HOMO_ were concentrated symmetrically around the oxygen atoms indicating areas able to donate electrons to unoccupied *d*−orbitals the metal surface, thus adsorbing and imparting corrosion inhibition [12]. Moreover, as seen in Figure 13c, the *E*_LUMO_ are mainly concentrated around the P atom indicating the ability of PO_4_^3−^ to receive electrons and creating feedback bonds. These two quantum properties are attributed to the chemisorption aspect of the inhibition mechanism where the sharing of electrons to the metal surface occurs. Figure 13d illustrates the electrostatic potential mapping of PO_4_^3−^ where red illustrates most negative regions while blue most positive. The negative regions are concentrated at the three oxygen atoms with lone pair electrons indicating that these areas of the ion are active sites of adsorption to the positively charged metal surface–electrostatic interactions (i.e., physisorption). This can be further elucidated using the atomic Mulliken charges found in Table 7, where most negative excess charges were found at the same oxygen atoms with unpaired electrons, making them nucleophilic reagents of the adsorption process forming the FePO_4_ protective passive film [36]. Corrosion inhibitors in different environments are presented in Table 8 along with their corresponding concentrations, *IE*, *E*_HOMO_, *E*_LUMO_, and Δ*E*_gap_. As it can be seen, the Δ*E*_gap_ are relatively low ranging from 5.9 to 8.64 eV, which can be used as a measurement of the corrosion inhibitor reactivity. However, the Δ*E*_gap_ is not the only factor in determining the effectiveness of the inhibitor, since the corrosion inhibition process is a complex interaction of several properties (electronic, chemical, and physical) between the molecule and the metal surface.

## 4. Conclusions

The corrosion inhibition properties of Na_3_PO_4_ were thoroughly investigated using different electrochemical tests, surface characterizations, and DFT calculations for carbon steel samples in 0.6 M Cl^−^ contaminated SCPS at 25 °C. It was concluded that Na_3_PO_4_ is an effective, environmentally friendly, and cheap anodic corrosion inhibitor that is able to achieve an *IE* up to 91.7% by forming a double-layered passive film where the inner layer is made of Fe_2_O_3_ and/or Fe_3_O_4_ and an outer layer of insoluble ferric phosphate (FePO_4_). It was found through Mott−Schottky analysis that the formed film was more orderly and less defective compared to the blank. This phenomenon was corroborated through the EIS film thickness calculations (*d*_eff,film_). The *d*_eff,film_ was found to be lower in the presence of Na_3_PO_4_ inhibited samples, indicating the formation of a densely packed and compact passive film, which could be attributed to an interphase inhibition mechanism. Na_3_PO_4_ followed the Langmuir adsorption isotherm having a Δ*G*^°^_ads_ of −22 kJ/mol indicating that the adsorption mechanism is a complex mix between physisorption and chemisorption. Dissociated phosphate ions will compete with Cl^−^ to adsorb on the surface of the carbon steel sample. Once adsorbed, PO_4_^3−^ will react with ferrous ions creating ferrous phosphate (Fe_3_(PO_4_)_2_) which will eventually be oxidized to insoluble ferric phosphate (FePO_4_); this was elucidated using FT−IR and XPS. Finally, DFT calculations were conducted to understand the strong inhibition performance of Na_3_PO_4_. It was found that the dissociated PO_4_^3−^ had a low Δ*E*_gap_ which can contribute to the inhibition process.

## Figures and Tables

**Table 1:** Elemental composition of carbon steel samples used for electrochemical testing (wt.%).

C	Mn	P	S	Si	Cu	Ni	Cr	Mo	V	Fe
0.28	1.08	0.019	0.043	0.20	0.37	0.16	0.16	0.050	0.0379	Bal.

**Table 2:** PDP curves electrochemical parameters for carbon steel samples in the presence and absence of different concentrations of Na_3_PO_4_ at 25 °C in 0.6 M Cl^−^ SCPS.

	[Na_3_PO_4_] (M)	*E*_corr_ (mV_SSC_)	*i*_corr_ (µA cm^−2^)	*IE * (%)	*θ*	*β*c (mV/dec)	*β*a (mV/dec)
Blank	-	−514	5.20	-	-	190	189
Na_3_PO_4_	0.05	−390	0.43	80.8	0.808	194	86
	0.10	−341	0.50	87.5	0.875	194	75
	0.30	−378	0.65	90.4	0.904	211	122
	0.60	−371	1.00	91.7	0.917	242	396

**Table 3:** EIS fitting data results for carbon steel samples in the absence and presence of different concentrations of Na_3_PO_4_ in 0.6 M Cl^−^ SCPS at 25 °C.

	[Na_3_PO_4_] (M)	*R*_s_ (Ω cm^2^)	*R*_film_ (Ω cm^2^)	*Y*_film_ (S cm^−2^ s^nfilm^)	*n*_film_	*R*_ct_ (Ω cm^2^)	*Y*_dl_ (S cm^−2^ s^ndl^)	*n*_dl_	*IE* (%)	*χ*^2 (^*^)^
Blank	-	6.22	4.80 × 10^2^	4.35 × 10^−6^	0.95	3.27 × 10^3^	2.71 × 10^−5^	0.78	-	6.64 × 10^−4^
Na_3_PO_4_	0.05	5.98	7.68 × 10^2^	4.58 × 10^−6^	0.96	1.04 × 10^4^	2.77 × 10^−5^	0.76	68.6	5.50 × 10^−4^
	0.10	8.64	1.10 × 10^3^	5.96 × 10^−6^	0.90	1.45 × 10^4^	5.47 × 10^−6^	0.83	77.4	1.34 × 10^−4^
	0.30	8.89	1.92 × 10^3^	6.42 × 10^−6^	0.91	2.23 × 10^4^	1.55 × 10^−6^	0.90	85.4	5.54 × 10^−4^
	0.60	8.46	2.69 × 10^3^	6.84 × 10^−6^	0.91	2.86 × 10^4^	4.17 × 10^−6^	0.80	88.6	5.47 × 10^−4^

* Total Error < 10%.

**Table 4:** The *C*_eff,dl_, *C*_eff,film_, and *d*_eff,film_ for blank and Na_3_PO_4_ inhibited carbons steel samples at different concentrations in 0.6 M Cl^−^ SCPS at 25 °C.

	[Na_3_PO_4_] (M)	*C*_eff,dl_ (F cm^−2^)	*C*_eff,film_ (F cm^−2^)	*d*_eff,film_ (nm)
Blank	-	2.38 × 10^−6^	4.47 × 10^−6^	5.92
Na_3_PO_4_	0.05	1.75 × 10^−6^	4.92 × 10^−6^	5.39
	0.10	7.12 × 10^−7^	7.15 × 10^−6^	3.71
	0.30	4.47 × 10^−7^	7.88 × 10^−6^	3.36
	0.60	3.21 × 10^−7^	8.76 × 10^−6^	3.03

**Table 5:** Adsorption isotherms used along with their regression coefficient [37].

Adsorption Isotherm	*R* ^2^	Equation
Langmuir	0.999	$\frac{C_{\mathrm{inh}}}{\theta }=\frac{1}{K_{\mathrm{ads}}}+C_{\mathrm{inh}}$
Temkin	0.866	$e^{\left(-2a\theta \right)}=K_{\mathrm{ads}}C_{\mathrm{inh}}$
Freundlich	0.912	$\theta =K_{ads}C_{\mathrm{inh}}^{n}$
Frumkin	0.920	$\log\left(C_{\mathrm{inh}}\frac{\theta }{1-\theta }\right)=2y\theta +2.303\log\left(K_{\mathrm{ads}}\right)$
El-Awady	0.932	$\log\left(\frac{\theta }{1-\theta }\right)=y\log\left(C_{\mathrm{inh}}\right)+\log\left(K_{\mathrm{ads}}\right)$

**Table 6:** Different chemical quantum properties calculated for PO_4_^3−^ ion using DFT.

*E*_HOMO_ (eV)	*E*_LUMO_ (eV)	Δ*E*_gap_ (eV)	*µ*_D_ (Debye)
−2.88	5.76	8.64	1.79

**Table 7:** Mulliken charge distribution for PO_4_^3−^ ion.

Atom	O1	O2	O3	O4	P
Mulliken charge	−1.212	−1.323	−1.323	−1.323	2.182

**Table 8:** Chemical quantum properties of corrosion inhibitors and their inhibition efficiency [56,57,58,59].

Corrosion Inhibitor	Environment	Substrate	Concentration	*IE* (%)	*E*_HOMO_ (eV)	*E*_LUMO_ (eV)	Δ*E*_gap_ (eV)	Ref.
Polymethacrylic acid	0.3 M Cl^−^ SCPS	Carbon steel	1 wt.%	71.51	−7.56	−1.39	6.17	[56]
Polymethacrylic acid-*co*-2-Acrylamido-2methylpropane sulfonic acid	0.3 M Cl^−^ SCPS	Carbon steel	1 wt.%	87.96	−7.36	−1.46	5.90	[56]
Potassium Sodium Tartrate	0.5 M Cl^−^ SCPS	Carbon steel	0.1 M	87.20	−8.11	−1.48	6.63	[57]
Sodium Acetate	0.5 M Cl^−^ SCPS	Carbon steel	0.125 M	81.00	−7.87	−0.48	7.38	[57]
1-ethyl-3-methylimidazolium tetrafluoroborate	1 M HCl	Mild Steel	500 ppm	82.41	−8.29	−1.40	6.89	[58]
N-Methyl-N,N,N-trioctylammonium chloride	1 M HCl	Mild steel	4.95 µM	93.20	−6.06	0.04	6.10	[59]
PO_4_^3−^ (Na_3_PO_4_)	0.6 M Cl^−^ SCPS	Carbon steel	0.6 M	91.70	−2.88	5.76	8.64	Present study

## Data Availability

The raw/processed data required to reproduce these findings cannot be shared at this time as the data also form part of an ongoing study.
